# Differential Age-Based Response Induced by a Commercial Probiotic Supplementation in Pastured Goats

**DOI:** 10.1007/s12602-024-10337-w

**Published:** 2024-08-05

**Authors:** Eunice Ndegwa, Doaa E. Elhadedy, Caitlyn Richey, Chyer Kim, Adnan B. Yousuf

**Affiliations:** 1 Agricultural Research Station, Virginia State University, Petersburg, VA USA; 2 Temple University, Philadelphia, PA USA

**Keywords:** Age-based, Effects, Pastured goats, Probiotics, Response

## Abstract

The potential benefit of probiotics in small ruminant production systems has largely been unexplored. We evaluated the effect of a goat commercial probiotic on health and performance indicators in pastured goats from birth until 10 months. We randomly allocated 26 newborn nursing goat kids to two groups: a control group that received saline and a treatment group that received a commercial probiotic paste orally. We evaluated select observable health indicators (inappetence, diarrhea, coughing), weight, immunity (IgA, IgG, and innate immune response), total protein, hematocrit (HCT), total lactic acid bacteria (LAB), total coliforms, and prevalence of *Escherichia coli* (*E. coli*) primary virulence genes (*stx1*, *stx2*, and *eae*) during the experimental period. The results revealed no significant differences in the health indicators, LAB count, and total *E. coli* count. Prevalence of *stx1* at 1 week of age and both *stx1* and *stx2* genes 4 months post-weaning was significantly (*P* < 0.05) higher in probiotic-supplemented goats. Probiotic supplementation significantly (*P* < 0.05) increased the total protein and IgA 1 month post-supplementation during the pre-weaning period and innate immune markers 2 days post-weaning. The HCT in probiotic-supplemented goats was significantly (*P* < 0.05) higher at 1 and 2 months post-weaning. The growth rate was not affected by probiotic supplementation in pre- and peri-weaned goats but was significantly (*P* < 0.05) lowered in goats older than 4 months in the supplemented group. In this pastured goat production study, there were mixed responses to a commercial probiotic in healthy goats based on age. The study suggests that early daily probiotic supplementation in pre-weaned pastured goats may have immune stimulation benefits, but in older healthy animals, post-weaning net benefits are unclear and further research is recommended.

**Supplementary Information:**

The online version contains supplementary material available at 10.1007/s12602-024-10337-w.

## Introduction

Impaired gut health due to diarrhea associated with bacterial, viral, and parasitic infections causes significant losses and reduced profitability in young small ruminants [1, 2]. Young animals before and around weaning are most vulnerable but heavily parasitized animals of any age often succumb to opportunistic bacterial infections [3, 4]. In very young kids and lambs (< 2 weeks), bacterial diarrhea commonly referred to as scours occurs independent of parasitic infections [5]. Control of bacterial diarrhea in young ruminants involves administration of fluid therapy and when indicated, broad-spectrum antibiotics [6]. Due to the widespread scourge of antimicrobial resistance, there is a global call for a reduction of the use of antimicrobials in animals [7–9] and finding alternatives to antibiotics [10]. There is also an increased consumer demand for naturally or organically produced products which calls for increased research for alternatives to antibiotics in animals [11–13]. Among the most widely researched alternatives for antibiotics are probiotics [10, 14, 15]. Probiotics are microorganisms generally regarded as safe (GRAS) that have beneficial effects when ingested by a host. Most of these microorganisms are bacteria although some yeasts are also found to possess beneficial health qualities in humans and animals [16, 17]. Probiotics are effective in controlling diarrhea, improving performance, and controlling food safety pathogens in food animals [17, 18]. The mechanisms of action of probiotics in animals are thought to include enhanced mucosal immunity, competitive exclusion of pathogenic organisms, and in other cases production of toxic metabolites that affect pathogenic organisms [19, 20]. In addition, probiotics also help in establishing a healthy microbiota in the gut and enhance gut tissue maturation and integrity in young animals [21–23].

There is scarcity of data on benefits of probiotics in small ruminants targeting the different production and management systems and also age-related responses. Recent scientific arguments have highlighted the complexity of the probiotic effects claims used by marketing agencies to attract end users. These underscore the fact that probiotic effect in the target hosts may not be uniform but may be affected by many other factors including diet, species of animal, individual animal/human resident gut microflora, and age among others [20, 24–26] subsequently calling for research on precision probiotics [26]. On the other hand, some commercial probiotics are marked for use in all ages of animals, without consideration of the different production systems and diets available for animals including age-based feeding differences. Given the different production and management systems in which farm animals are reared and costs incurred in buying feed supplements, there is a need to evaluate potential probiotic benefits for each system and age groups to clearly define where benefits are achievable. In some markets like the US, few commercial goat-specific probiotics are available some of which are broadly recommended for all ages.

Therefore, in this study, we evaluated the influence of probiotic supplementation beginning early in life of pastured goat kids and during the growing period on health parameters, immune markers, microbial populations, and performance.

## Material and Methods

### Study Animals and Husbandry

The study was carried out at Virginia State University (VSU), USA, small ruminant research unit. The goat flock is comprised of myotonic and Spanish breeds that are bred in November and kid in March/April yearly. A cohort of 26 newborn goat kids were recruited into the study and followed until 10 months of age. The goat kids (19 myotonics and 7 Spanish) included on the study were born on pasture end of March and first week of April and were assigned randomly to the treatment groups. Only goat kids that appeared healthy and nursing at the beginning of the experiment were selected for inclusion in the experiment. These remained with their nursing does on pasture until weaning at approximately 3 months old. All experimental animals were vaccinated against clostridial diseases (CD & T) at 35 days post-treatment (35 dpt). Thereafter, the goats and goat kids remained on pasture with daily supplementation with a corn-soybean ration at 2% body weight. Baled hay was supplemented as needed in winter at 7 and 8 months post-weaning (7 and 8 mpw). The pasture is mainly composed of eastern gamagrass (*Tripsacum dactyloides*) pasture with volunteer common Bermuda grass (*Cynodon dactylon*) and herbaceous annual grass legumes. All experimental animals were managed similarly and remained together until the end of the experiment. Animals were monitored daily for signs of illness (diarrhea, inappetence, and coughing) or death. Animals were evaluated for signs of parasitemia using Famacha scores [27] and diarrhea during each fecal collection and dewormed accordingly. Each health issue, mortality, and/or deworming event was recorded. Animals were weighed weekly during the pre-weaning and peri-weaning periods (1 week post-weaning) and monthly thereafter. Animals were cared for according to an approved Virginia State University Institutional Animal Care and Use Research Protocol (VSU AACUC #2018-001).

### Probiotic Supplementation

The study hypothesis was that an early gut supplementation with beneficial probiotic microorganisms would establish a healthy microbiome, boost immunity, and result in better performance in supplemented animals. Consequently, probiotics were started at two days of birth and supplemented daily for the first month (28 days). Thereafter, probiotics were supplemented weekly until the day of weaning. Consideration was also given to the most stressful period (weaning period); thus, from the weaning day until 1 week post-weaning, probiotics were again supplemented daily. Post-weaning, probiotics were supplemented monthly to boost healthy bacterial populations in the gut (Table 1). The commercial probiotic used in the study (Goats Prefer^®^ Probiotic Plus Paste, Vets Plus, Inc, Menomonie, WI, USA) is marketed in the US for goats of all ages with dosage ranges indicated on the tube. In this study, the dosages used are shown in Table 1. The viable microorganisms indicated in the tube include *Lactobacillus acidophilus*, *Enterococcus faecium*, *Lactobacillus plantarum*, and *Lactobacillus casei* (grouped as lactic acid bacteria), 5 billion colony forming units (CFU).

**Table 1:**
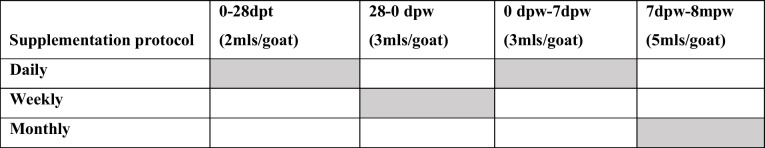
Probiotic supplementation protocol

*dpt* days post-treatment, *dpw* days post-weaning, *mpw* months post-weaning

### Fecal Sample Collection

Fecal samples (*n* = 563) were first collected within 48 h (2 days) pre-supplementation, followed by weekly samplings until the day of weaning (0 dpw), 2 days post-weaning (2 dpw), 1 week post-weaning (7 dpw), and thereafter monthly until 10 months of age. At each sampling, individual fecal samples were collected from the rectum either by a moistened swab (2, 7, 14, and 28 days) or by a lubricated gloved finger rectally (all other samplings). Fecal samples were subsequently transported in ice to the laboratory for microbial enrichment, microbial isolation, and parasite microscopy.

### Blood Sample Collection and Processing

Blood samples (*n* = 333) were collected from the jugular vein bi-weekly during the pre-weaning period and monthly post-weaning. HCT was determined using 75-mm hematocrit tubes (Kimble^®^, Rockwood, TN, USA) and an LW Scientific USA E8 Series (Lawrenceville, GA) centrifuge following the manufacturer’s whole blood spinning protocol and the accompanying EZ Reader for hematocrit values. Serum was extracted by allowing the blood to clot at room temperature for an hour followed by centrifugation at 1500 rpm for 15 min. The serum was stored at − 80 °C until further processing for immune markers and total protein.

### Microbial Enrichment, Isolation, and Identification of Lactic Acid Bacteria in Fecal Samples and Probiotic Paste

Fecal samples were subjected to an initial 24-h enrichment in tryptic soy broth (TSB) (MP Biomedicals, Solon, OH, USA) (for *E. coli* enrichment) or 48-h enrichment under anaerobic incubation in De Man, Rogosa, and Sharpe (MRS) broth containing Tween 80 (MilliporeSigma, Billerica, MA, USA)) (for LAB enrichment). Two hundred milligrams (200 mg) of solid fecal sample or 200-µl fecal solution was added to 3 ml TSB or MRS broth media. Part of the enrichment was used for total DNA extraction and the rest was stored in 20% glycerol at – 80 °C until further analysis.

The commercial probiotic paste (three randomly selected tubes) was also subjected to MRS enrichment to confirm the presence of live LAB as indicated on the tube. Briefly, a loopful of the probiotic paste was transferred to 3-ml sterilized MRS broth media and incubated anaerobically at 37 °C for 48 h. Serial dilutions of the resulting growth were prepared up to 10^6^ and 20 µl from each dilution plated on MRS agar followed by incubation at 37 °C for 48 h. The presence of live bacteria was confirmed by the presence of colonies on the MRS plates.

### Microbial DNA Extraction from TSB and MRS Enrichment

Extraction of total DNA from enrichment followed a simple boiling method. Briefly, 1–2 ml of the enrichment broth was centrifuged in a Heraeus Fresco 21 centrifuge (Thermo Scientific, Waltham, MA, USA) at 10,000 rpm for 3 min to pellet bacteria in the sample. The pellet was washed twice with 1-ml molecular-grade water. The supernatant was poured off, and 200-µl fresh molecular-grade water was added to the pellet followed by resuspension through vortexing. The suspension was heated at 100 °C for 10 min in a Digital Dry Bath (USA Scientific, Ocala, FL, USA), followed by centrifugation for 4 min at 14,800 rpm to pellet microbial contents. One hundred and fifty microliters (150 µl) of the supernatant (containing DNA) was transferred to a clean tube, and DNA concentration was measured by Nanodrop 2000 (Thermo Scientific,Waltham, MA, USA).

### LAB and *E. coli* Abundance Quantification Using PCR

Quantification of LAB was done by SYBR green qPCR protocol using the 16 s ribosomal RNA gene primers of LAB described in [28]. A standard curve was generated using PCR products from the amplification of ATCC 4356 DNA and primers F: 5-AGCAGTAGGGAATCTTCCA-3 and R5, CACCGCTACACATGGAG. The *E. coli Uid A* gene primers [29] were used for *E. coli* quantification. Extracted DNA was diluted to between 10 and 100 ng of DNA. A standard curve was generated using PCR products generated from the amplification of the ATCC 25922 *Uid A* gene. The samples were run in duplicate, and a cycle threshold (Cq) was used to calculate and quantify the log count of LAB or *E. coli* in each sample using the standard curve generated. Evaluation of the melting curve was used to confirm the amplification of each bacterium sp in each sample. The limit of detection for both LAB and *E. coli* was 10 genome copies. For all bacterial detection and quantification, the amplification protocol followed the Applied Biosystems PowerUp™ SYBR Green Master Mix reaction setup recommendations except for the annealing and extension temperature that was unique for the primer pair used in this study. The total reaction volume was 10 µl for all reactions. The annealing temperature for *E. coli* was 56 °C for 15 s followed by an extension of 72 °C for 1 min; quantification data were collected at both the annealing and extension steps. *Lactobacillus* sp annealing temperature was 58 °C for 15 s, extension at 72 °C for 30 s, and final extension at 80 °C for 30 s with data collection being at 80 °C followed by a melting curve analysis cycle. The qPCR program was 40 cycles for both bacteria using a QuantiStudio 3 PCR system (Thermo Scientific, Waltham, MA, USA).

### Detection of *E. coli* Virulence Genes Using Multiplex PCR

Detection of *E. coli* virulence gene was carried out using DNA extracted from an overnight TSB fecal sample enrichment as described above. The protocol used was a previously published multiplex PCR targeting the *E. coli* shiga toxin genes *stx1* and *stx2* and *eae* using gene-specific primers [30] and the Appendix. The reaction was carried out in a 25 µl total volume using 12.5 µl DreamTaq PCR master mix (Thermoscientific™, Waltham, MA), 0.25 µl of each primer, 100 ng DNA, and molecular-grade water. The PCR protocol included an initial denaturation at 95 °C for 3 min followed by 40 cycles of 95 °C for 30 s, annealing at 55 °C for 30 s, extension at 72 °C for 1 min, and a final extension at 72 °C for 5 min. Amplified products were run on a 1.5% thidium bromide gel followed by visualization under UV light in a Gel Imager (Applied Biosystems).

### Hematological Analysis

Sera were evaluated for total IgA, IgG, total serum protein, and mixed antibody/complement immune response analysis using commercially available assay kits. Analysis for IgG and IgA utilized kits and antibodies from Bethyl Laboratories (Fortis Life, MA, USA) following the manufacturer’s recommendations. Mixed antibody/complement immune responses to probiotic supplementation were evaluated using a simple rapid immunity test D2Dx according to manufacturer instruction Nano Discovery Inc. (Orlando, Florida). Total protein was determined using a Reichert^®^ Vet360-Chek (Depew, New York) digital refractometer. Samples were diluted 1:10 for IgG and IgA using recommended kit diluents and carried out in duplicates.

### Fecal Microscopic Evaluation

In all animals with diarrhea and high Famacha scores, fecal floatation using magnesium sulfate (400 g/l) solution was carried out using the procedure described in the Cornell University website (http://goatdocs.ansci.cornell.edu/CSGSymposium/BasicQuantitativeFecalExaminationMethod.pdf). Measurement of the fecal sample and volume of flotation solution were modified based on the amount of fecal sample available for each animal. The presence of *Coccidia* and *Strongyle* eggs was evaluated using a microscope (Optika Microscope, Ponteranica BG, Italy) at × 10 magnification and recorded.

### Statistical Analysis

Individual animals were considered experimental units. Data was analyzed using *t*-test (GraphPad Software© *t*-test calculator) (https://www.graphpad.com/quickcalcs/ttest1/) comparing values for control and probiotic-supplemented goats. Differences were considered significant at *P* < 0.05. The proportion of animals with *E. coli* virulence genes was compared using MedCalc’s comparison of proportion calculator. (https://www.medcalc.org/calc/comparison_of_proportions.php).

## Results

### Health Indicators and Growth Performance

**Fig. 1:**
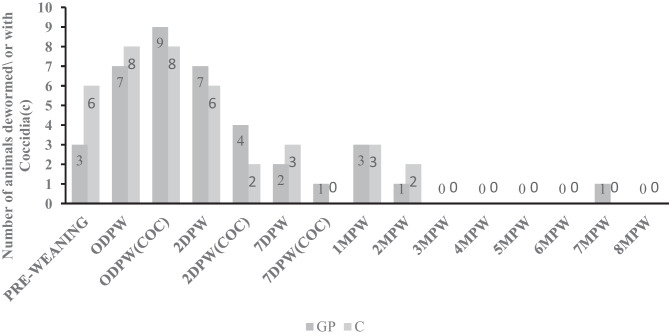
Number of animals dewormed or with coccidia during the experimental period

No negative health effects were observed in animals supplemented with probiotics as compared to non-supplemented animals. Three animals were lost from the experiment from each group due to non-treatment-related deaths (predation, injuries). Some animals experienced parasite-related diarrhea from both experimental groups mostly during the pre-weaning and peri-weaning periods. In addition, some animals from both groups had high Famacha scores (> 3) indicating infestation by *Haemonchus contortus*. Both groups were dewormed as needed with a combination of Cydectin^®^ and Levamisole^®^ or Valbazen^®^ drenches based on the weight. Figure 1 shows the number of animals dewormed and with detectable coccidia in fecal samples from each experimental group throughout the study period. No significant differences were detected in the number of animals dewormed or with diarrhea or coccidia detected in both groups during the indicated periods. When comparing the deworming or parasite presence between the growing periods (pre-weaning vs peri-weaning (0 dpw, 2 dpw,7 dpw) vs post-weaning (1–8 mpw)) as the figure shows, there was a significant (*P* < 0.05) number of animals dewormed and with coccidia during the peri-weaning period compared to the other growth period irrespective of the treatment group.

**Fig. 2:**
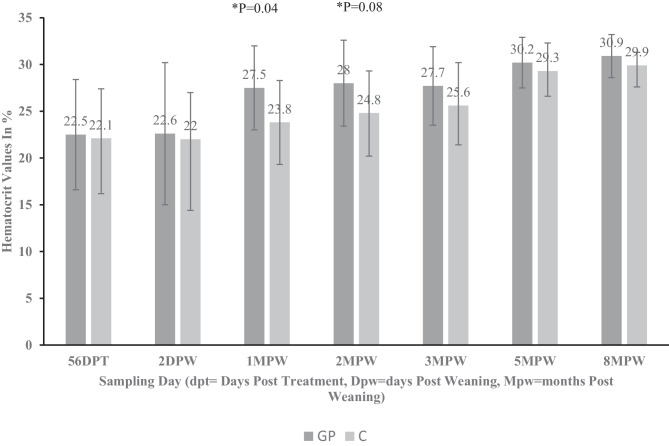
Hematocrit values in goats supplemented with a commercial probiotic (GP) and non-supplemented group (C)

The hematocrit was determined as a complementary health indicator, especially concerning parasitism by *H. contortus*, coccidian, and other internal blood-sucking worms. Values were determined at around 2 months of age to 6 months post-weaning representing the critical growing period when animals are susceptible to parasitism (Fig. 2). Blood was also collected at slaughter (8mpw) from five representative animals from each treatment group. Figure 2 shows the mean hematocrit values recorded for the different treatment groups. The values tended to be similar before weaning 56 dpt and peri-weaning (2 dpw). However, 1-month post-weaning (1 mpw), the hematocrit values in the probiotic-supplemented group was significantly (*P* = 0.04) higher than the control group and remained higher in this group 2 months after weaning (2 mpw, *P* = 0.08). Overall, the hematocrit values were slightly higher for the probiotic-supplemented goats post-weaning until the end of the experiment.

### Growth Performance

**Fig. 3:**
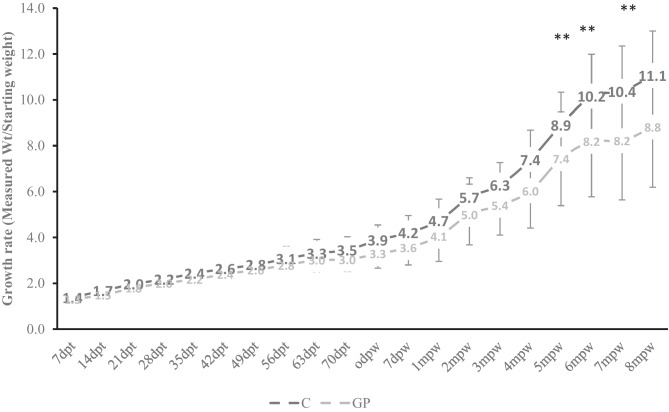
Comparison of growth rate in probiotic-supplemented vs control pastured goats from 2 days post-birth to 8 months after weaning (**P < 0.05)

The growth rate was calculated based on the mean weight at each sampling, the mean weight gain, and average daily gain (ADG) at different growth periods (representing the different probiotic treatment regimens) (Table 1) and as a fraction of birth weight (growth rate, Fig. 3). The mean starting goat kid weight at 2 days old was comparable between the two treatment groups (6.4 lbs vs 6.5 lbs for probiotic-supplemented vs control animals respectively). The mean weight and the growth rate for both groups followed the same pattern with a peak growth rate detected during the post-weaning period (0 dpw–6 mpw). During the pre-weaning period and peri-weaning, as shown in Fig. 3, there were no significant differences in the growth rate between the two treatment groups. However, during the more rapid growth rate period after weaning (2–8 mpw), the growth rate for the control group tended to be higher than the probiotic-supplemented group: 3 mpw, 4 mpw, and 5 mpw. Beyond 5 mpw, the growth rate for control goats was significantly (*P* < 0.05) higher than the probiotic-supplemented group at 6, 7, and 8 mpw.

As shown in Table 2, the mean weight gain and average daily gain (ADG) recorded during pre-weaning and peri-weaning for the control group were slightly higher than those for the probiotic-treated group. On the contrary, during the period 1-week post-weaning (7 dpw) to 2 months post-weaning (2 mpw), the mean weight gain for the probiotic group was slightly higher than that for the control group although the differences were not significant (Table 2). Thereafter, the mean weight gain for the control group was higher than that for the probiotic-supplemented group and was significantly (*P* = 0.018) different between 4 and 5 mpw (Table 2).

**Table 2:** Mean weight gain and average daily gain at different growth periods

Treatment period	Control wt (SEM)	Probiotic wt (SEM)	*P*-value
0 dpt–28 days post-trt			
Mean weight gain (lbs)	7.6 (0.41)	6.7 (0.38)	*P* = 0.14
ADG	0.27	0.24	
28 dpt-weaning			
Mean weight gain (lbs)	11 (1.08)	9.1 (1.06)	*P* = 0.25
ADG	0.21	0.17	
Peri-weaning (0–7 dpw)			
Mean weight gain (lbs)	2 (0.39)	1.7 (0.7)	*P* = 0.77
ADG	0.28	0.25	
7 dpw–1 mpw			
Mean weight gain (lbs)	3.2 (0.67)	3.8 (0.98)	*P* = 0.64
ADG	0.14	0.16	
1–2 mpw			
Mean weight gain (lbs)	6 (0.4)	6.7 (0.38)	*P* = 0.25
ADG	0.2	0.22	
2–3 mpw			
Mean weight gain (lbs)	3.4 (0.7)	3.1 (0.73)	*P* = 0.8
ADG	0.11	0.1	
3–4 mpw			
Mean weight gain (lbs)	7.1 (1.16)	6.6 (0.88)	*P* = 0.75
ADG	0.24	0.22	
4–5 mpw			
Mean weight gain (lbs)	9.6 (0.92)	6.6 (0.63)	*P* = 0.018**
ADG	0.32	0.22	
5–6 mpw			
Mean weight gain (lbs)	8.4 (1.84)	5.9 (1.9)	*P* = 0.34
ADG	0.28	0.2	
6–7 mpw			
Mean weight gain (lbs)	1.5 (0.75)	0.5 (0.67)	*P* = 0.21
ADG	ND	ND	
7–8 mpw			
Mean weight gain (lbs)	4.5 (0.6)	4.5 (0.6)	*P* = 1
ADG	0.2	0.2	

*dpt* days post-treatment, *dpw* days post-weaning, *mpw* months post-weaning, *SEM* standard error of the mean, *ND* Not done, *Wt* weight, *ADG* average daily gain, *trt* treatment, *lbs* pounds

### Total Serum Protein and Immune Parameters

**Fig. 4:**
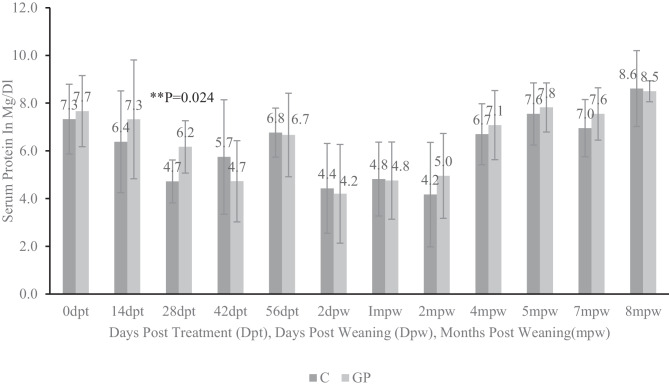
Total protein comparison between probiotic-supplemented and non-supplemented pastured goats

Figure 4 shows the total serum protein values in the two groups of experimental goats. Animals were vaccinated against clostridial diseases at 35 days post-initiation of the experimental treatment (35 dpt). Total serum proteins were highest at 8 mpw and lowest at 2 dpw. After 14 days (14 dpt) of daily probiotic supplementation, the total serum protein readings for probiotic-treated goat kids were higher but not significantly different from the control group. However, after 28 days of probiotic supplementation (28 dpt), goats receiving probiotics had significantly higher total protein readings than goats in the control group. At 42 days post-treatment (42 dpt), a much lower reading was recorded for the probiotic-supplemented group compared with control goats which coincided with 1 week after clostridial vaccine (CDT) was administered to the goat kids. In subsequent samplings, no differences were detected between the treatment groups.

**Fig. 5:**
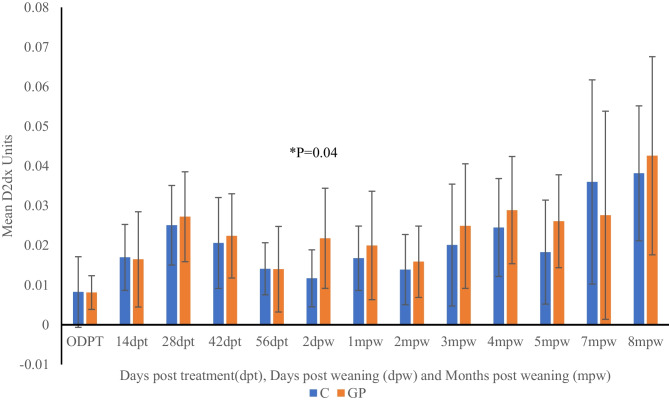
Comparison of total immune stimulation

Changes in combined immune health values as measured by the D2Dx kit for both probiotic-supplemented and control animals were highest at 8 mpw and lowest on the first day of sampling (0 dpt) (Fig. 5). During the pre-weaning period, values rose to the highest at 28 dpt declining to lowest values at 2 dpw for the control animals and 56 dpt in the probiotic-supplemented animals. However, there were no significant differences between probiotic-supplemented and control goats during the pre-weaning period up to 56 days post-treatment. On the other hand, 2 days after weaning (2 dpw), the probiotic-supplemented goats had significantly higher immune values than the non-supplemented groups (*P* = 0.04). Post-weaning, except at 7 months post-weaning (7 mpw), the values for probiotic-supplemented goats were higher, but there were no significant differences between the groups.

**Fig. 6:**
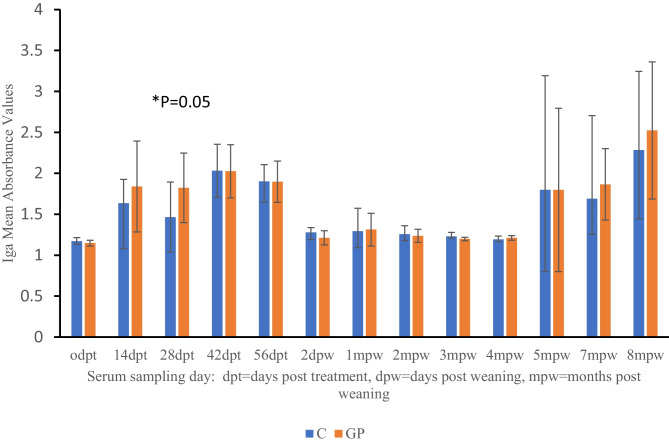
Comparison of relative total IgA values

Comparison of antibody-mediated immune modulation between probiotic-supplemented and control goats was evaluated using the total IgG and IgA absorbance values in each set of serum samples collected at each sampling point. There were no differences in the IgG mean absorbance values between the treatment groups detected (data not shown). The mean absorbance values for IgA were slightly higher for goats receiving probiotic supplements compared to controls 2 weeks after the supplementation began and were significantly different after 28 days of supplementation (*P* = 0.05) (Fig. 6). No differences were detected from 56 days post-treatment between the treatment groups. At 5, 7, and 8 mpw, there was high variability in individual animal IgA absorbance values although the mean absorbance values for probiotic-supplemented goats were higher at 7 and 8 mpw.

### Total LAB and *E. coli* Counts

*E. coli* and LAB were detected in all age groups at all sampling times (Supplementary Data). For both groups of bacteria, higher counts were detected before and around the weaning period. The highest and lowest counts of LAB were detected at 7 dpt and 8 mpw respectively, but no significant differences in LAB counts were detected between the treatment groups at any time. The highest *E. coli* counts were detected 1 week post-weaning while the lowest counts were detected 1 and 4 months after weaning. *E. coli* counts detected at 42 dpt and 2 and 7 dpw in probiotic-treated groups tended to be higher compared to controls although the differences were not significant.

### Prevalence of *E. coli* Virulence Genes

Primary *E. coli* virulence genes including shiga toxins *(stx1* and *stx2*) and intimin protein gene (*eae*) were detected in the majority of the experimental goat kids at the beginning of the experiment before supplementation of the probiotic treatment (Table 3). The highest number of animals harboring *E. coli* virulence genes was detected during the pre-weaning period (0–56 dpt) followed by the peri-weaning period (0–7 dpw) while the lowest number was detected at 7 mpw. At 1 week of age (7 dpt) and 4 months after weaning(4 mpw), the number of goat kids with the *stx1* gene detected was significantly higher in the probiotic-treated group of goat kids (*P* < 0.05) compared to controls. Likewise, at 4 mpw, the number of goat kids with the *stx2* gene detected was significantly higher (*P* = 0.04) for the probiotic-treated group. The most prevalent virulence genes during the pre-weaning period were *eae*, *stx1*, and *stx2* in descending order irrespective of the treatment group.

**Table 3:** Prevalence of three *E. coli* primary virulence genes in probiotic-supplemented and control goat kids (t1, control; t2, probiotic treated)

Days post-treatment/weaning dpt/dpw	Treatment groups	# with virulence gene	*Stx1*	*Stx2*	*eae*
0 dpt	t1 (*n* = 12)	10	5	2	9
	t2 (*n* = 14)	10	3	4	7
7 dpt	t1 (*n* = 10)	9	3*	3	8
	t2 (*n* = 12)	12	7**	3	7
14 dpt	t1 (*n* = 11)	10	9	3	9
	t2 (*n* = 11)	10	10	5	8
21 dpt	t1 (*n* = 10)	6	4	0	3
	t2 (*n* = 12)	10	6	4	4
28 dpt	t1 (*n* = 10)	8	4	4	5
	t2 (*n* = 11)	8	7	5	2
56 dpt	t1 (*n* = 11)	10	5	6	2
	t2 (*n* = 13)	8	5	7	3
0 dpw	t1 (*n* = 11)	6	3	2	3
	t2 (*n* = 11)	8	4	4	3
2 dpw	t1 (*n* = 11)	7	1	3	1
	t2 (*n* = 8)	5	3	5	0
7 dpw	t1 (*n* = 11)	5	1	4	4
	t2 (*n* = 11)	8	3	7	5
1 mpw	t1 (*n* = 10)	5	4	5	3
	t2 (*n* = 10)	6	2	6	0
4 mpw	t1 (*n* = 9)	2	1*	0^a^	2
	t2 (*n* = 10)	5	5**	4^b^	0
6 mpw	t1 (*n* = 8)	4	2	2	2
	t2 (*n* = 10)	5	1	2	3
7 mpw	t1 (*n* = 9)	1	0	1	0
	t2 (*n* = 9)	0	0	0	0

*/**Values are significantly different

^a,b^Values are significantly different

## Discussion

In this study, mixed responses were detected, and the effect of commercial probiotic supplementation was different depending on the age of the goats. A number of beneficial effects were detected during the pre-weaning growth period while both positive and negative effects were detected in post-weaned goats. Probiotics improved total IgA and total proteins during the first month of daily probiotic supplementation. These benefits were not associated with any significant increase in LAB counts in probiotic-supplemented goat kids suggesting the improved immune benefits may not be due to increased LAB but rather other modes of immune modulation. Recent reviews have highlighted studies that show that ability to establish in the target host may not be critical to induce some health benefits and that non-viable probiotic products are also capable of stimulating the immune system and inducing beneficial health outcomes [31–33]. The beneficial effect of probiotics in enhancing immunity in host animals is well documented [34–36]. In this study, we demonstrate for the first time in pastured nursing goat kids supplemented with a commercial probiotic, this benefit was achievable. This is significant since most gut health-related disease conditions occur in this age group of goats [37, 38], especially in poorly managed or intensively managed flocks [39]. For these types of production systems, the findings suggest that probiotic supplementation in this age group may be an option to boost immunity and potentially offer resistance against pathogens. Few studies have evaluated the immune-modulating effect of probiotics in young goat kids. In one study in Taiwan [40], *Enterococcus faecium* SF68 supplementation improved immunoglobulins and total proteins in young goat kids similar to this study although the latter study goat kids were maintained on a commercial milk replacer instead of nursing. In agreement with this study findings, probiotics also improved IgA, IgG, IgM, IFN-y, IL-2, and total proteins in growing female Beichuan white goats [41]. In a study involving 5-month-old lambs, probiotic supplementation with a yeast-based preparation resulted in increased IgA, IgG, and IgM values [42]. Similar improvements in IgA and IgG have been reported in other young ruminants including one study in pre-weaned calves supplemented with a multispecies probiotic mixture [43, 44] and both serum proteins and total immunoglobulins in another multispecies probiotic supplementation in calves in China [45]. The higher serum proteins detected in probiotic-supplemented goat kids in this study during the pre-weaning period did not translate to superior growth rate. Notably, higher serum proteins coincided with the period when higher IgA was detected in supplemented animals. Since the total serum protein includes both albumin and globulins, it may be that the higher total protein detected was due to immune stimulation–associated proteins rather than growth stimulation. On the other hand, some studies reported no antibody-related immune benefit in pre-weaned calves [46] or increased serum proteins [47]. The difference may be due to a variety of factors including differences in the type and strain of probiotic used, host species–specific responses, and/or doses supplemented. The latter study used *Bacillus subtilis natto* which differs from the ones found in the commercial probiotic used in this study; (mixed *Lactobacillus*, *Pediococcus*, and *Enterococcus* species). These findings further emphasize the complexity and highly variable probiotic responses in host species that are dependent on multitude of factors. Probiotic supplementation did not affect the growth performance in pre-weaned goats and decreased performance post-weaning in this study. Studies evaluating the effect of probiotics on growth performance in small ruminants are scarce [48]. In a previous study with post-weaned goats, the authors, in agreement to the current study finding, reported no clear growth performance benefit of supplementing a commercial probiotic in several experiments [49]. Similarly, in another recent study with growing female goats, no improvement in weight was detected [41]. In contrast, another study involving creole goats reported improved performance and meat fatty acid profile following supplementation with a mix of probiotics [54]. In the latter study, the authors used native probiotic species previously isolated from healthy goat feces. In another study involving growing Maltese goats, improved weight gain was reported post-supplementation with a mixture of probiotics [55]. Studies with other ruminants also report differing outcomes of performance indicators after probiotic supplementation. In pre-weaned calves in two studies, no significant increase in weight in probiotic-administered calves compared to control animals [45, 50] was detected. Similarly, in one lamb study, no improvement in performance was detected in probiotic-supplemented pre-weaned lambs but there was a tendency to show a modest increase post-weaning [51]. Many studies in poultry and swine, however, report improved growth performance (reviewed in [52, 53]). Some other studies involving pre-weaned calves reported improved growth performance post-supplementation with probiotics [44, 47, 54]. Due to the different experimental designs including species of animals, age of animals, production systems, and species and doses of microorganisms in the probiotic preparations, it is difficult to fully explain differences or similarities detected in different probiotic animal studies. The cause of decreased growth rate in post-weaned animals receiving probiotics in this study is unclear. One possibility could be that probiotic species in the supplement may have caused disruptions in microbial homeostasis in the rumen or intestines of the otherwise healthy experimental animals in this study.This could occur especially if the source of the microorganism is not the native species in the goat and or the age group. Recent publication showing clear age-based differences in the predominant native LAB species in pastured goats could offer insight into this hypothesis [55]. Since ruminants heavily depend on ruminal microbes for nutrition, any changes in structure may have resulted in altered gut metabolism and affected growth [19]. Another interesting finding was the average daily gain (ADG) in the first 2 months post-weaning that was slightly higher in probiotic-supplemented goats than in control goats, which coincided with significantly higher hematocrit in the treatment groups compared to controls. Probiotics had some health-protective benefits in supplemented goats during this immediate post-weaning period probably due to improved hematocrit that may have helped animals infected by *H. contortus* be more resilient to the negative effects. Causes of differences in the response to probiotics in animals have been extensively described and may include the animal species, diet, age, health state, type of probiotics, and doses source of antibiotics [19]. Some of these factors could have played a role in the differential responses detected in this study.

This study reported an improved HCT post-weaning in goats supplemented with probiotics. Similar improvements in HCT post-probiotic supplementation have been reported in many other species of animals, for example, in calves supplemented with yeast and *Enterococcus* spp [56], sheep [57], rabbits [58], fish [59–61], turkeys [62], and chickens [63]. In contrast, some studies reported a decreased HCT in fish post-probiotic supplementation [64] and no effect in chickens [65] and cattle [66]. The mechanisms involved in improved hematocrit after probiotic supplementation have not been evaluated but are desirable effects in host animals especially in goats infected by blood-sucking parasites like *H. contortus*.

Lactic acid bacteria (LAB) and *E. coli* were detected in both probiotic-supplemented and control goats but did not differ significantly at any time during the experimental period. For both groups of bacteria, the highest counts were detected during the pre-weaning period, and the lowest counts were detected in mature goats after weaning. The lack of significant differences in lactic acid bacteria counts between probiotic-supplemented and control goats could be due to many factors. One possibility could be due to competition from resident microflora of the healthy animals or failure of the administered probiotic strains to establish in the experimental animals. This phenomenon has recently been reviewed as it applies to human probiotic consumption revealing that probiotic establishment and effect in the host vary greatly and some individual gut microbiomes may resist colonization [25]. This has led to a call for personalized and precision probiotic development as opposed to empirical probiotics [26, 67]. The probiotics used in this study did not indicate the host or age group of source of the probiotics used in the commercial preparation. A recent longitudinal study characterizing LAB in pastured goats revealed age-based diffrences in abundance and diversity with high prevalence and diverse species of native LAB in pre-weaned goats and very low prevalence in goats over 1 month old [55]. Interestingly, in the latter pasture study, the strains of LAB detected in the different age groups were different and the most common strains used in probiotic preparations, *Lactobacillus* and *Pediococcus* species, were predominantly detected in pre-weaned animals. Whether this implies that mature goat gastrointestinal tract may not support establishment of these species remains to be investigated. Nevertheless, in the same study, some unique LAB strains were detected in a few mature goats that with further analysis may reveal if any probiotic or health benefits are associated with their presence in the adult animals. A recent study comparing the effect of host-specific and non-host-specific probiotics in pigs revealed superior performance of host-specific probiotics in enhancing the health of the pigs [68]. Similarly, using a commercial probiotic in previous studies with goats, no differences in lactic acid bacteria were detected between supplemented and control goats [49] or in humans [69] and in chicken [70]. Probiotic supplementation did not also reduce the *E. coli* counts at any time during the experiment in this study. Zhang and Kim, on the other hand, found decreased *E. coli* and increased *Lactobacillus* spp in a multistrain probiotic-supplemented vs control chicken [71]. *E. coli* belonging to the STEC remain relevant as a potential human pathogen but those carrying the intimin gene (*eae*) and enteropathogenic *E. coli* (EPEC) also cause diseases in a wide host of animal species including ruminants [72]. An important application of probiotics in animals is for reduction of pathogenic bacteria including *E. coli* strains especially during stressful periods when animals are at higher risk of succumbing to infections [73]. Animals harboring *E. coli* virulence genes (*stx1*, *stx2*) STEC and *eae* genes were detected in both supplemented and control goats throughout this study albeit in a higher proportion of animals during the pre-weaning compared to the post-weaning period, an observation previously reported in a longitudinal study [74]. After 1 week of probiotic supplementation and 4 months after weaning (4 mpw), more goats supplemented with probiotics had *stx1* and both *stx1* and *stx2* respectively than control goats in the absence of any observed differences in *E. coli* counts. The reason behind this observed difference in virulence genes in this study is not clear but certainly warrants further investigation. A potential explanation may be probiotics in this preparations interrupted the normal gut microflora in the otherwise healthy goat kids causing microbial dysbiosis that resulted in proliferation of the STEC. Although no studies were found evaluating effects of probiotics on STEC prevalence in goats, varying results ranging from no effect to reduction of pathogenic *E. coli* by probiotic supplementation have been reported in other species of animals. A few examples include a reduction in STEC in sheep [75] and cattle [76, 77], a reduction in pathogenic *E. coli* in chickens [78], no effect in chickens [79], and no effect in cattle [80, 81]. Although many studies have evaluated benefits of probiotics in animals and humans, many gaps still remain in understanding the causes of the variable probiotic effects reported in different studies. In animal studies, consideration of age, diet, species, and also production and management system among others are factors that need to be considered in future development of precision probiotics.

## Conclusion

This work reports the effect of a commercial probiotic in pastured goats highlighting differential responses observed in the different age groups. While probiotics showed immune benefits in pre-weaned and peri-weaned animals, no overall improvement in growth performance indicators was realized at any stage of growth as a result of probiotic supplementation. Nevertheless, these early growth benefits may translate to improved performance under some production and management systems. Mixed effects were reported in older animals including a negative effect observation on growth rate that call for further research. Whether these findings are due to the type of microorganisms in the preparation, production, or management system of the goats remains to be explored. The results do however point to the controversial question of the universalities of the commonly marketed probiotics to all age groups, benefits across multiple species, and no regard to husbandry system in animals. Thus, the results call for further research on the beneficial effects of probiotics in different ages of target species, different husbandry systems, and the health status of animals. Furthermore, the study results underscore the recent call by many experts for the development of individualized, host-specific, age-adapted probiotic products that may be the future of probiotic application in human and animal medicine.

## Electronic supplementary material

Below is the link to the electronic supplementary material.

Supplementary file1 (PPTX 108 KB)

## Data Availability

All data is available from corresponding author upon request.

## Appendix

Shiga toxin and intimin protein gene primers used in the study

- ***stx1-F*** TGT CGC ATA GTG GAA CCT CA; ***stx1-R*** TGC GCA CTG AGA AGA AGA GA (655 bp)
- ***stx2-R*** TGT CGC CAG TTA TCT GAC ATT C; ***stx2-F*** CCA TGA CAA CGG ACA GCA GTT (477 bp)
- ***eae-F*** CAT TAT GGA ACG GCA GAG GT; ***eae-R*** ACG GAT ATC GAA GCC ATT TG (375 bp)
