# Supplementary figures and images for: Differential Age-Based Response Induced by a Commercial Probiotic Supplementation in Pastured Goats
Source: Probiotics Antimicrob Proteins. 2024 Aug 5;17(6):3960–74. doi: 10.1007/s12602-024-10337-w (PMC12634735; doi:10.1007/s12602-024-10337-w)

## Slide 1
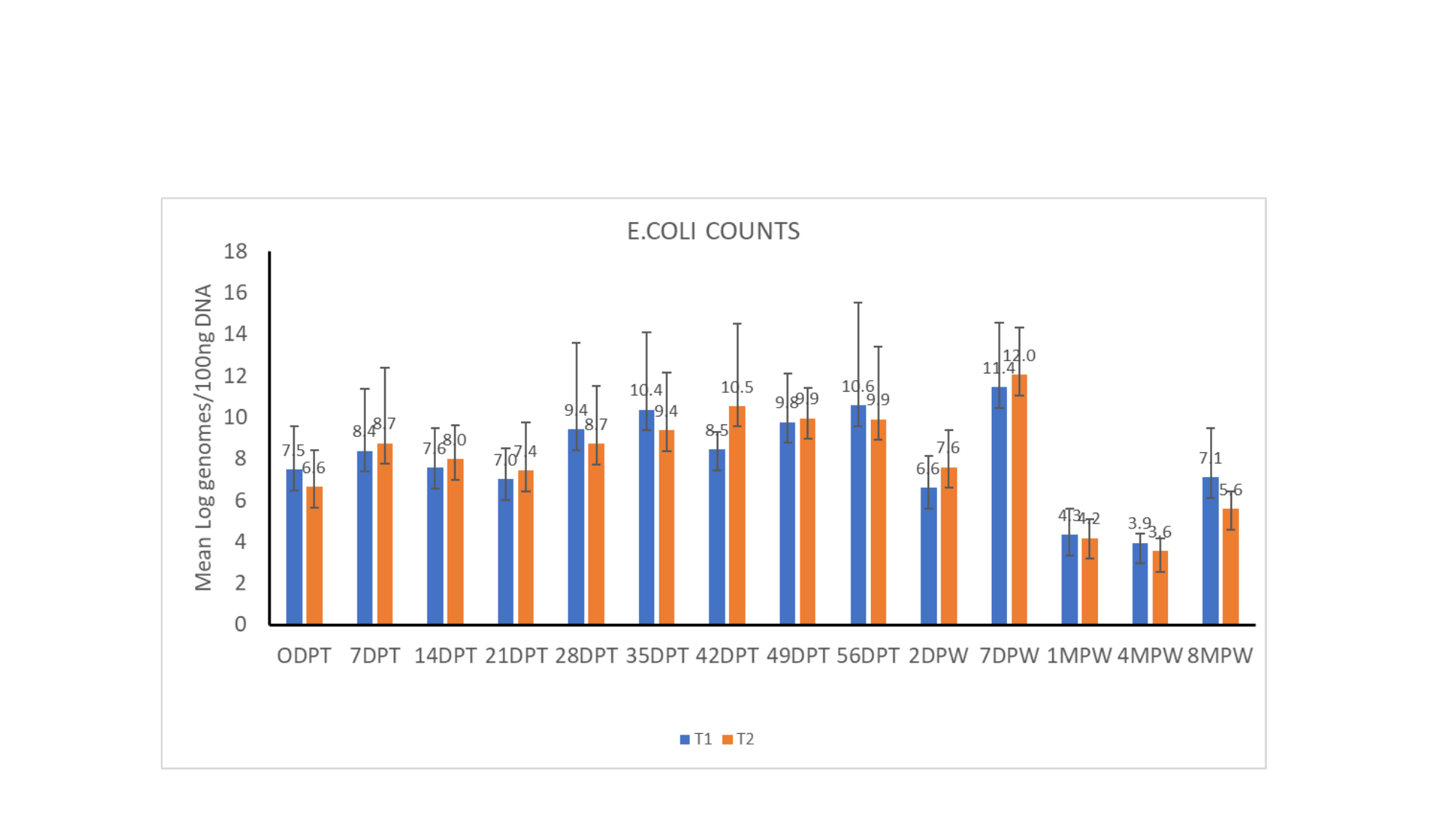

## Slide 2
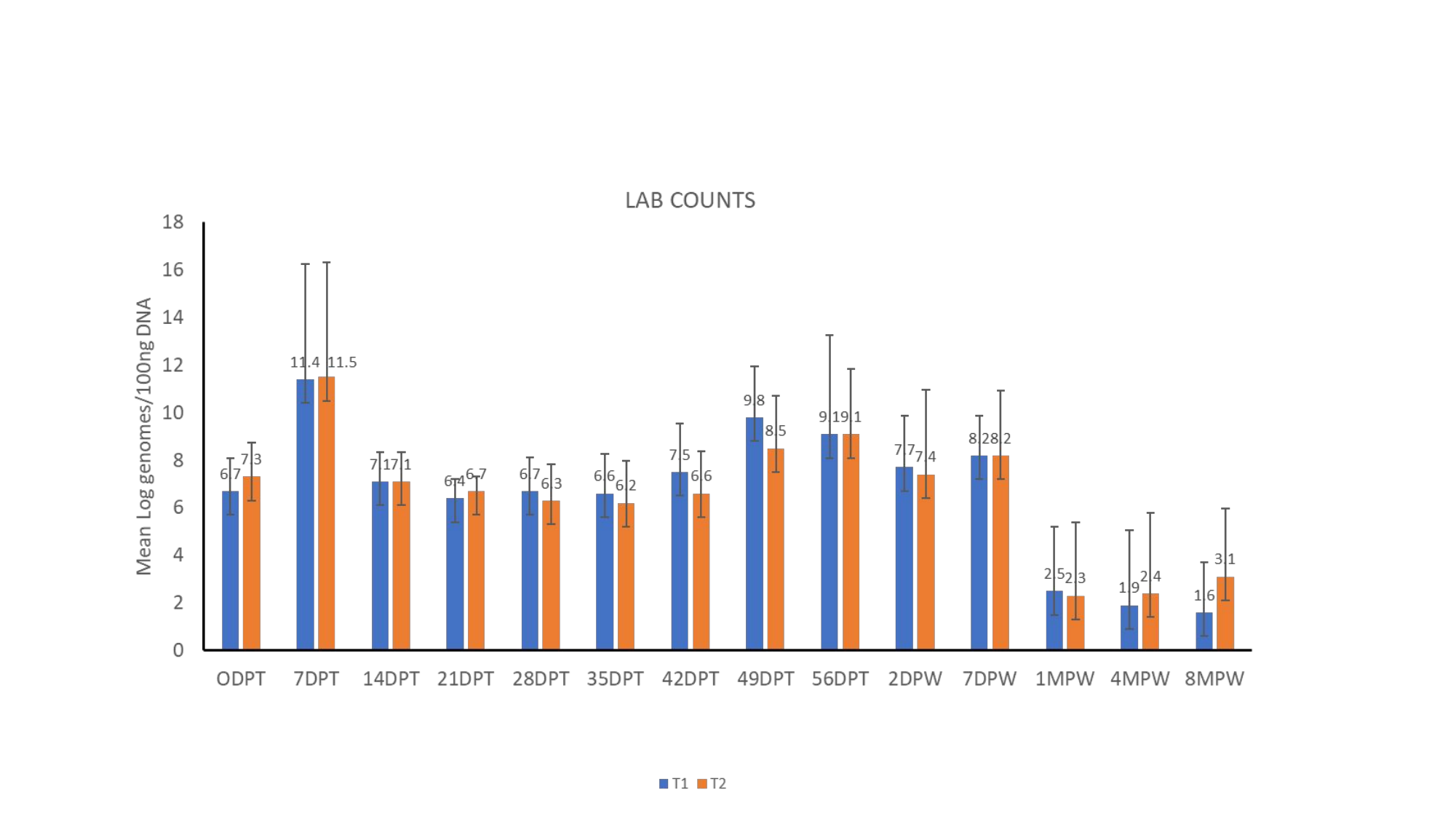

Supplement: Supplementary file 1 — Supplementary file1 (PPTX 108 KB) [file 12602_2024_10337_MOESM1_ESM.pptx]
